# Chemical composition and tyrosinase inhibitory activity of *Cinnamomum cassia* essential oil

**DOI:** 10.1186/1999-3110-54-10

**Published:** 2013-08-21

**Authors:** Chen-Tien Chang, Wen-Lun Chang, Jaw-Cherng Hsu, Ying Shih, Su-Tze Chou

**Affiliations:** 1 Department of Food and Nutrition, Providence University, 200 Chung-Chi Road, Shalu, Taichung, 43301 Taiwan, R.O.C; 2 Department of Cosmetic Science, Providence University, 200 Chung-Chi Road, Shalu, Taichung, 43301 Taiwan, R.O.C; 3 Department of Applied Cosmetology, Master Program of Cosmetic science, HungKuang University, 34 Chung-Chi Road, Shalu, Taichung, 44302 Taiwan, R.O.C

**Keywords:** Cinnamaldehyde, *Cinnamomum cassia* Presl, Mixed-type inhibition, Mushroom tyrosinase, Tyrosinase inhibitor

## Abstract

**Background:**

Essential oils extracted from aromatic plants exhibit important biological activities and have become increasingly important for scientific research. The essential oil extracted from *Cinnamomum cassia* Presl (CC-EO) has various functional properties, however, little information is available regarding the tyrosinase inhibitory activity. Therefore, the objectives of this study were to investigate the chemical composition and tyrosinase inhibitory activity of the CC-EO.

**Results:**

*cis*-2-methoxycinnamic acid (43.06%) and cinnamaldehyde (42.37%) were found to be the two major components of the CC-EO identified by gas chromatography–mass spectrometry (GC-MS). The inhibitory activities of CC-EO and its major constituents were further evaluated against mushroom tyrosinase. The results showed that CC-EO and cinnamaldehyde exhibited anti-tyrosinase activities with IC_50_ values of 6.16 ± 0.04 mg/mL and 4.04 ± 0.08 mg/mL, respectively. However, cis-2-methoxycinnamic acid did not show any anti-tyrosinase activity. The inhibition kinetics were analyzed by Lineweaver-Burk plots and second replots, which revealed that CC-EO and cinnamaldehyde were mixed-type inhibitors. The inhibition constants (Ki) for CC-EO and cinnamaldehyde were calculated to be 4.71 ± 0.09 mg/mL and 2.38 ± 0.09 mg/mL, respectively.

**Conclusion:**

These results demonstrate that CC-EO and its major component, cinnamaldehyde, possess potent anti-tyrosinase activities and may be a good source for skin-whitening agents.

**Electronic supplementary material:**

The online version of this article (doi:10.1186/1999-3110-54-10) contains supplementary material, which is available to authorized users.

## Background

Tyrosinase (monophenol, dihydroxyphenylalanine: oxygen oxidoreductase EC 1.14.18.1) is a multifunctional, copper-containing enzyme that is widely distributed in nature and is involved in melanogenesis. It catalyzes both the hydroxylation of L-tyrosine (monophenolase activity) and the oxidation of L-DOPA (diphenolase activity) to *o*-quinone, which induces the production of melanin pigments (Seo et al. 2003). Melanin formation is considered to be deleterious to color quality and results in a loss of nutritive and market values for plant-derived foods and beverages (Friedman 1996; Sánchez-Ferrer et al. 1995). In addition, excessive melanin accumulation leads to human skin disorders, such as melasma, freckles, age spots and malignant melanomas (Fitzpatrick et al. 1983). Furthermore, formation of neuromelanin in the mammalian brain may be related to neurodegeneration associated with Parkinson’s disease (Asanuma et al. 2003). Therefore, the development of safe and effective tyrosinase inhibitors has become important for improving food quality and preventing pigmentation disorders and other melanin-related human health issues (Seo et al. 2003). Additionally, tyrosinase inhibitors are supposed to have broad applications as cosmetics whitening agents (Dooley 1997). As plants are a rich source of bioactive chemicals that are mostly free from harmful side effects, interest in finding natural tyrosinase inhibitors in bioactive chemicals is also increasing. Some potent tyrosinase inhibitors, such as anisaldehyde, quercetin and recently dalenin have been isolated from various plants (Chen and Kubo 2002; Chiari et al. 2011; Kubo and Kinst-Hori 1998).

*Cinnamomum cassia* Presl is widely cultivated in China. The dried stem bark of *C. cassia*, i.e., cassia bark, is important not only as a food spice but is also considered to have medicinal properties, such as antimicrobial (Lee and Ahn 1998), antitumorigenic (Ka et al. 2003), anti-inflammatory (Lee et al. 2002) and antidiabetic properties (Verspohl et al. 2005). In addition, the methanol extract of twigs of *C. cassia* was found to possess tyrosinase inhibitory activity (Ngoc et al. 2009). It is known that plant essential oil (EO) has various functional properties, such as a pleasant aroma, insect and animal repellant, as well as inhibitory effects against microorganisms. Moreover, various products made from EO have been used in aromatherapy and may relax or stabilize some physical and psychological conditions (Matsuura et al. 2006). The anise oil and citrus essential oils have been reported to possess tyrosinase inhibitory activities (Kubo and Kinst-Hori 1998; Matsuura et al. 2006). The essential oil from the relative cinnamon species, *C. zeylanicum* Blume, has been reported to show anti-tyrosinase activity, and cinnamaldehyde was found to be mainly responsible for this inhibition effect (Marongiu et al. 2007). The *C. cassia* essential oil (CC-EO) has hypouricemic (Zhao et al. 2006) and antifungal activities (Zhao et al. 2006). A number of tyrosinase inhibitors from both natural and synthetic sources that inhibited monophenolase, diphenolase or both of these activities have been identified to date (Kim and Uyama 2005). These tyrosinase inhibitors include plant polyphenols and aldehydes, fungal metabolites, derivatives of natural compounds, and synthetics origins. The tyrosinase inhibitory constituents of the essential oil extracted from *Cinnamomum cassia* (Ngoc et al. 2009; Lee et al. 2002) and relative cinnamon species, *C. zeylanicum* (Marongiu et al. 2007) have been well documented. Cinnamaldehyde was found to be the major constitute of the essential oil. However, the inhibitory pattern of cinnamaldehyde isolated from *C. cassia* (Lee et al. 2002), *C. zeylanicum* (Marongiu et al. 2007), olive oil (Kubo and Kinst-Hori 1999) and the root of *Pulsatilla cernua* was rather controvesial. Cinnamaldehyde from *C. cassia* was a competitive tyrosinase inhibitor whereas those from *C. zeylanicum*, olive oil and and the root of *Pulsatilla cernua* was a noncompetitive tyrosinase inhibitor. Therefore, this study were to further investigate the chemical composition and tyrosinase inhibitory activity of the CC-EO, including identifying constituent compounds using GC-MS, determining IC_50_ values using inhibition kinetic analysis, and determining tyrosinase inhibitory patterns and inhibition constants using Lineweaver-Burk plots and second replots. In addition, the results were compared with those of the well-known tyrosinase inhibitor kojic acid.

## Methods

### Chemicals

Mushroom tyrosinase (EC 1.14.18.1; T7755), *trans*-cinnamaldehyde, *cis*-2-methoxycinnamic acid and kojic acid were purchased from Sigma-Aldrich Chemicals Co. (St. Louis, MO, U.S.A). L-3,4-Dihydroxyphenylalanine (L-DOPA) was purchased from Merck Co. (Darmstadt, Germany). All other chemicals were reagent grade or higher.

### Essential oils

The essential oil, which was obtained by steam distillation from the stem bark of *C. cassia* Presl, was purchased from Yangsen Biotech, Inc. (Taipei, Taiwan). The extraction procedure is according to previous study with slight modifications (Choi et al. 2001). Briefly, *C. cassia* stem bark was place into vessel and extracted by steam distillation for 4 hours. The vapors were cooled by a closed cooling system and the liquid were collected in a container. The oils floated towards the top while the water settled below and the essential oils were obtained by simply removing the oils which were separated.

### Enzymatic assay of tyrosinase

Tyrosinase inhibitory activities of the CC-EO and its major constituents were determined by the tyrosinase-dependent L-3,4-dihydroxyphenylalanine (L-DOPA) oxidation assay according to a slight modification of the method of Kubo and Kinst-Hori (1998). The substrate solution (0.84 mL of 0.89 mM L-DOPA in 16 mM sodium phosphate buffer, pH 6.8) was incubated at 25°C for 10 min. Following incubation, 0.03 mL of each sample solution and 0.03 mL of mushroom tyrosinase (1000 units/mL; T7755, Sigma, one unit = ΔOD_280_ of 0.001 per min at pH 6.5 at 25°C in 3 mL reaction mixture containing L-tyrosine) were added. The assay mixture in a total volume of 0.9 mL was immediately monitored for the formation of dopachrome by measuring the linear increase in optical density at 475 nm. Kojic acid, which is known to inhibit tyrosinase (Chen et al. 1991a), was used as a positive control. The inhibitory percentage of tyrosinase was calculated as follows: % inhibition = (1-B/A) × 100, where A = ΔOD_475_/min without tested sample and B = ΔOD_475_/min with tested sample. The 50% inhibition (IC_50_) of tyrosinase activity was calculated as the concentrations of the tested sample that inhibited 50% of tyrosinase activity under experimental conditions.

### Kinetic analysis

The reaction mixture consisted of 0.3 mL of L-DOPA (0.75-3 mM) as a substrate, 0.54 mL of 25 mM sodium phosphate buffer (pH 6.8), 0.03 mL of mushroom tyrosinase (1000 units/ml) in 25 mM sodium phosphate buffer (pH 6.8), and 0.03 mL of each sample solution (0–12.50 mg/mL for CC-EO; 0–6.25 mg/mL for *trans*-cinnamaldehyde) in a total volume of 0.9 mL was assayed at 25°C, as described above. The inhibitory kinetics of each sample with tyrosinase were analyzed using Lineweaver-Burk plots. The reciprocal equation for a rapid equilibrium approach from the mixed-type noncompetitive inhibition can be expressed as **eq 1** (Segel 1976). The *K*_i_ for CC-EO and cinnamaldehyde were calculated from the slope replots (**eq 2**). The *αK*_i_ for CC-EO and cinnamaldehyde were calculated from the 1/*v* axis intercept replots (**eq 3**). **Eq 1**, $\frac{1}{v}=\frac{K_{s}}{V_{\max}}\left(1+\frac{\left[I\right]}{K_{i}}\right)\frac{1}{S}+\frac{1}{V_{\max}}\left(1+\frac{\left[I\right]}{\alpha K_{i}}\right)$; **eq 2**, $\mathrm{Slope}=\frac{K_{s}}{V_{\max}}+\frac{K_{s}}{V_{\max}K_{i}}\left[I\right]$ and **eq 3**, 1vaxisintercept=1VmaxαΚiI+1Vmax, where *K*_s_ is the dissociation constant of substrate (S) from enzyme-substrate complex (ES), *K*_i_ is the dissociation constant of inhibitor (I) from enzyme-inhibitor complex (EI), and *αK*_i_ is the dissociation constant of inhibitor from enzyme-substrate-inhibitor complex (ESI).

### GC-MS analysis

GC-MS analyses were carried out on a GCMS-QP-2010 plus Gas chromatograph Mass Spectrometer (Shimadzu, Japan) and GCMS-solution software (v. 2.50 SU3, Shimadzu, Japan). Compounds were separated on a Forte ID-BPX5 cross-linked 5% phenyl - 95% methyl polysiloxane (30 m × 0.25 mm i.d., film thickness 0.25 *μ* m) capillary column (SGE, AU). The column was maintained at 50°C for 5 min after injection then programmed at 5°C/min to 150°C, then programmed at 10°C/min to 300°C. The injection volume was 1.0 μl of pure essential oil, with a split ratio of 1:100. Helium was used as the carrier gas at a constant flow-rate of 1.0 ml/min. Injector, transfer line and ion-source temperatures were 250, 230 and 250°C, respectively. MS detection was performed with electron impact mode at 70 eV ionization energy and 60 *μ* A ionization current, by operating in the full-scan acquisition mode in the 40–350 amu range. Compounds were identified by comparing the retention times and retention indices of the chromatographic peaks with those of authentic reference standards run under the same conditions. Peak enrichment on co-injection with authentic reference compounds was also carried out. The comparison of the MS fragmentation pattern with those of pure compounds and mass spectrum database search was performed by using the National Institute of Standards and Technology (NIST) MS spectral database (version 2005).

### Statistical analysis

All the assays to determine enzyme activity (i.e., the tyrosinase inhibitory effect of CC-EO, *trans*-cinnamaldehyde, *cis*-2-methoxycinnamic acid and kojic acid and the enzyme kinetics) were conducted at least three times with three different sample preparations. All data were expressed as mean ± standard deviation (S.D.). Analysis of variance (ANOVA) was performed using SPSS (SPSS Inc., U.S.A.). A one-way ANOVA and Scheffe test were used to determine the difference of means, and *p* < 0.05 was considered to be statistically significant.

## Results

### Chemical composition of CC-EO

The chemical composition of CC-EO was analyzed using GC-MS. The 16 constituent compounds identified, along with the retention times and Kovats indices, are listed in Table 1. Our results showed that the two major constituents of CC-EO were *cis*-2-methoxycinnamic acid (43.06%) and cinnamaldehyde (42.37%) and that the minor compounds were *o*-methoxycinnamaldehyde (5.11%), 1,2-dimethoxy-4-(3-methoxy-1-propenyl) benzene (2.05%), cinnamyl acetate (1.83%) and other compounds (1.25~0.16%) in the present study.

**Table 1:** **GC/MS analysis of the essential oil from** ***Cinnamomum cassia*** **Presl**

No	Name	Rt^a^	KI^b^	Area
1	Benzaldehyde	7.58	982	0.42
2	2,2,4,6,6-Pentamethylheptane	7.84	983	0.21
3	2,5,9-Trimethyldecane	9.10	1121	0.49
4	2,5-Dimethylundecane	9.49	1136	0.33
5	Phenylethyl alcohol	11.79	1175	0.29
6	Cinnamaldehyde	16.57	1189	42.37
7	3,4-Dimethoxyphenethyl alcohol	17.78	1514	0.79
8	Germacrene D	18.97	1515	0.32
9	*cis*-2-Methoxycinnamic acid	19.69	1546	43.06
10	Cinnamyl acetate	20.88	1589	1.83
11	Coumarin	20.99	1623	1.25
12	*o*-Methoxycinnamaldehyde	22.70	1745	5.11
13	*trans*-Caryophyllene	22.95	1832	0.43
14	1,2-Dimethoxy-4-(3-methoxy-1-propenyl) benzene	24.11	1946	2.05
15	2-Ethyl-5-propylphenol	24.78	1993	0.21
16	β-Phenethyl cinnamate	32.01	2041	0.16

^a^Retention time (min); ^b^Kovats index.

### Effect of CC-EO, *trans*-cinnamaldehyde and *cis*-2-methoxycinnamic acid on the activity of mushroom tyrosinase

**Figure 1:**
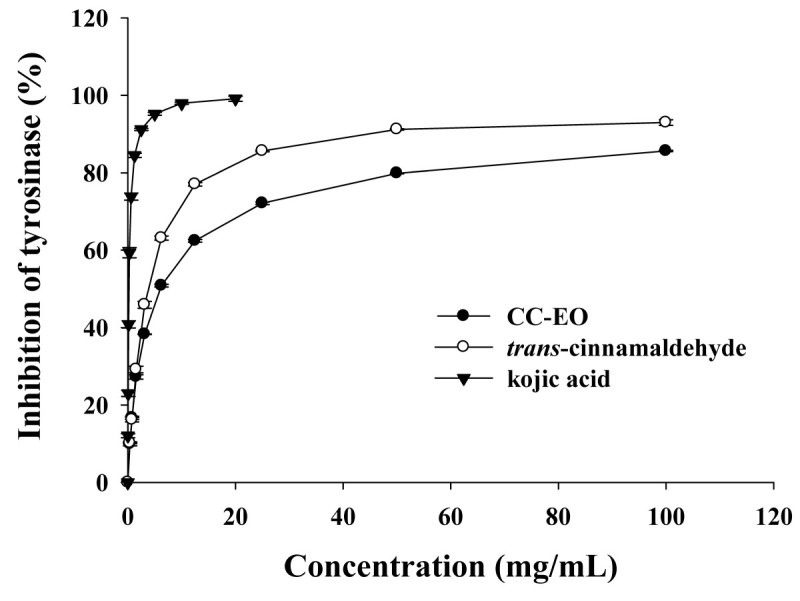
**Dose-dependent inhibition of mushroom tyrosinase by** ***C. cassia*** **essential oil (CC-EO),** ***trans*** **-cinnamaldehyde and kojic acid.** Tyrosinase activity was measured using L-DOPA as the substrate. Each value represents means ± S.D. (n = 3).

The effect of CC-EO, *trans*-cinnamaldehyde and *cis*-2-methoxycinnamic acid on the oxidation of L-DOPA catalyzed by mushroom tyrosinase as well as that of kojic acid which is a well-known tyrosinase inhibitor were studied. As shown in Figure 1, it was found that CC-EO and *trans*-cinnamaldehyde had potent inhibitory effects on the L-DOPA oxidase activity of mushroom tyrosinase in a dose-dependent manner. The results showed that *trans*-cinnamaldehyde had higher tyrosinase inhibitory activity than CC-EO. Based on the half-inhibition concentration (IC_50_) (Table 2), *trans*-cinnamaldehyde is approximately 1.5 times more effective than CC-EO. However, *cis*-2-methoxycinnamic acid had no inhibitory effects on the mushroom tyrosinase in this study (data not shown). Then, we had compared the tyrosinase inhibitory activity of the CC-EO with a well-known tyrosinase inhibitor, kojic acid. The IC_50_ value of the tyrosinase inhibitory activity of kojic acid was determined to be 0.22 mg/mL which was found to be significantly more pronounced than those of CC-EO (6.16 mg/mL) and *trans*-cinnamaldehyde (4.04 mg/mL). To obtain 80% tyrosinase inhibitory activity, the concentrations needed for CC-EO, *trans*-cinnamaldehyde, and kojic acid were 51.35, 16.65, and 0.96 mg/mL, respectively.

**Table 2:** **Inhibition constants of** ***C. cassia*** **essential oil (CC-EO),** ***trans*** **-cinnamaldehyde and** ***cis*** **-2-methoxycinnamic acid for mushroom tyrosinase**

Inhibitor	CC-EO	***trans***-cinnamaldehyde	***cis***-2-methoxycinnamic acid
IC_50_ (mg/mL)	6.16 ± 0.04^a*^	4.04 ± 0.08^*^	NS
*K*_i_ (mg/mL)	4.71 ± 0.09^*^	2.38 ± 0.09^*^	NT
*αK*_i_ (mg/mL)	15.50 ± 0.49^*^	9.61 ± 0.47^*^	NT
Inhibition type	Mixed	Mixed	NT

^a^Values are presented as mean ± S.D. (n = 3). NS: tyrosinase inhibitory effect not shown; NT: not tested.

^*^Means in the same parameter are significantly different (*p* < 0.05). *K*_i_: dissociation constant for inhibitor binding with the free enzyme; *αK*_i_: dissociation constant for inhibitor binding with the enzyme-substrate complex.

### Inhibition type of CC-EO and *trans*-cinnamaldehyde on the activity of mushroom tyrosinase

**Figure 2:**
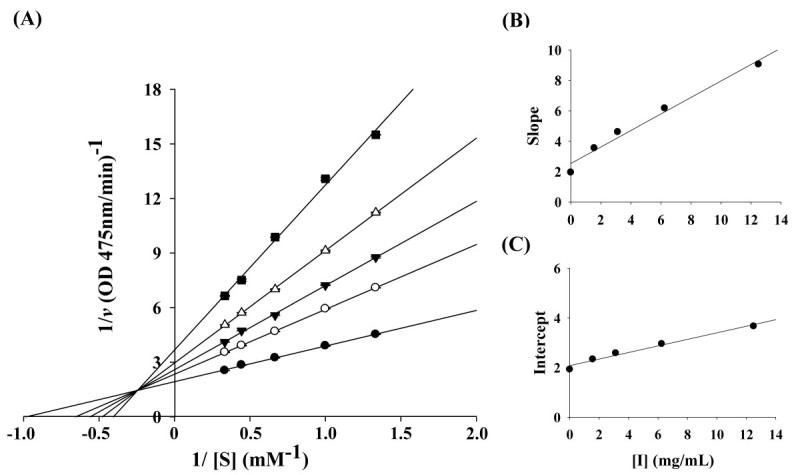
**Determination of inhibition type and inhibition constants of** ***C. cassia*** **essential oil. (A)** Lineweaver-Burk plots of mushroom tyrosinase and L-DOPA without (●) and with [(○) 1.56 mg/mL, (▼) 3.12 mg/mL, (△) 6.25 mg/mL and (■) 12.50 mg/mL] essential oil from *C. cassia*. **(B)** and **(C)** represent expressions of *K*_i_ and *αK*_i_, respectively.

**Figure 3:**
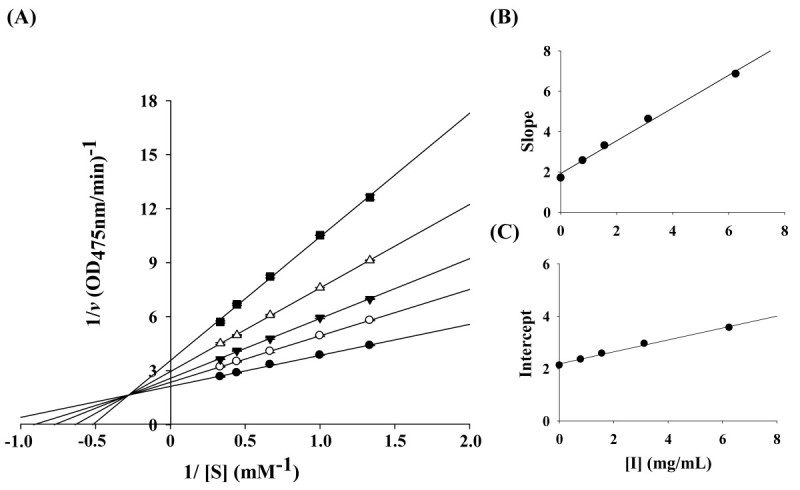
**Determination of inhibition type and inhibition constants of** ***trans-*** **cinnamaldehyde. (A)** Lineweaver-Burk plots of mushroom tyrosinase and L-DOPA without (●) and with [(○) 0.78 mg/mL, (▼) 1.56 mg/mL, (△) 3.12 mg/mL and (■) 6.25 mg/mL] *trans*-cinnamaldehyde. **(B)** and **(C)** represent expressions of *K*_i_ and *αK*_i_, respectively.

The kinetic behaviors of CC-EO, on the mushroom tyrosinase for the oxidation of L-DOPA were first studied. Under the experimental conditions, the Michaelis constant (*K*_*m*_) and maximum velocity (*V*_max_) of the L-DOPA oxidation reaction catalyzed by the tyrosinase (30 units) were 0.80 mM and 0.469 ΔOD_475_/min, respectively. The inhibitory kinetics of CC-EO were analyzed using Lineweaver-Burk double reciprocal plots as shown in Figure 2A. The five lines, obtained from the uninhibited enzyme and from four different concentrations of CC-EO, intersected to the left of the 1/*v* axis above the 1/*S* axis. Increased concentrations of CC-EO resulted in decreased *V*_max_ and an increased *K*_*m*_. These results indicate that CC-EO exhibited mixed-I type inhibition for the oxidation of L-DOPA catalyzed by mushroom tyrosinase. Similar results were obtained with *trans*-cinnamaldehyde (Figure 3A), showing that it was also a mixed-I type inhibitor for the enzyme. The dissociation constants for inhibitor binding with the free enzyme and the enzyme-substrate complex, *K*_i_ and *αK*_i_, were obtained from the double-reciprocal plots and the replots of the slope and the vertical intercept versus the concentration of CC-EO (Figure 2B, 2C**)** or *trans*-cinnamaldehyde (Figure 3B, 3C**)**, respectively. The obtained values are summarized in Table 2.

## Discussion

The essential oil extracted by steam distillation from the stem bark of *C. cassia* Presl was quantitatively analyzed by GC-MS. *cis*-2-Methoxycinnamic acid and cinnamaldehyde were determined as the major compounds of the oil (43.06% and 42.37%, respectively). For comparison, a previous study reported that cinnamaldehyde (92.2%) was the most plentiful constituent in the *C. cassia* essential oil (Giordani et al. 2006). Different extraction processes and assay methods could have contributed to differences in cinnamaldehyde levels of *C. cassia* essential oils (Dugoua et al. 2007). Cinnamaldehyde (77.1%) was also found to be the major constituent of volatile oil of the bark of *C. zeylanicum* (Marongiu et al. 2007). Several studies have demonstrated that cinnamaldehyde has antimicrobial (Ooi et al. 2006), antimutagenic (Shaughnessy et al. 2001), antitumorigenic (Ka et al. 2003) and immunomodulatory activities (Koh et al. 1998); furthermore, cinnamaldehyde is considered to possess tyrosinase-inhibitory effects with IC_50_ values from 0.52~0.97 mM (Lee 2002; Lee et al. 2000; Ngoc et al. 2009). Lee (2002) reported that 2-methoxycinnamic acid that had been isolated from *Pulsatilla cernua* root was a potent noncompetitive inhibitor of mushroom tyrosinase with an IC_50_ value of 0.34 mM*.* In addition, benzaldehyde, one of the flavor compounds characterized in anise oil, showed potent tyrosinase inhibitory activity with an IC_50_ of 0.82 mM (Kubo and Kinst-Hori 1998). Because the CC-EO contained major in cinnamaldehyde and *cis*-2-methoxycinnamic acid, and trace readings of benzaldehyde, however, there is no report directly evaluating the tyrosinase inhibitory activities of CC-EO.

Mushroom tyrosinase has been widely used as a target enzyme in screening and characterizing potential tyrosinase inhibitors. Because the inhibition mode depends on the structures of both the substrate and inhibitor, L-DOPA has been used as the substrate in this study. Therefore, the activity studied in this paper was the *o*-diphenolase inhibitory activity of mushroom tyrosinase. The results showed that CC-EO and its major constituent, cinnamaldehyde, showed a dose-dependent anti-tyrosinase effect, however no activity was observed for the other major constituent, *cis*-2-methoxycinnamic acid. Because CC-EO contains 42.37% of cinnamaldehyde, it is suggested that cinnamaldehyde is responsible for the tyrosinase inhibitory activity of CC-EO. Although pure *cis*-2-methoxycinnamic acid had no tyrosinase inhibitory activity in our study, Lee (2002) reported that 2-methoxycinnamic acid that had been isolated from *Pulsatilla cernua* root was a potent noncompetitive inhibitor of mushroom tyrosinase with an IC_50_ value of 0.34 mM. The discrepancy of these results may be due to different assay methods or inhibitor purity. The methanolic extracts of *C. cassia* or the bark essential oil from the different cinnamon species obtained using different extraction techniques such as supercritical CO_2_ fluid extraction have been reported to exert anti-tyrosinase activity (Marongiu et al. 2007; Ngoc et al. 2009). However, the extraction methods or plant species mentioned in these studies are different from the present paper. Moreover, the biological activities of plant extracts and essential oils are contributed to their bioactive components which may be affected by seasons, geographical origin, harvest time, agronomic practices and extraction methods (Fiocco et al. 2011). Kojic acid, a fungal secondary metabolic product produced by species of *Aspergillus* and *Penicillium*, was shown to inhibit mushroom tyrosinase activity (Chen et al. 1991b). Kojic acid has been extensively used as a medical agent for the treatment of a number of different skin disorders associated with hyperpigmentation. To reach a similar degree of tyrosinase inhibitory effect, the concentration required for CC-EO or its major constituent, cinnamaldehyde, was significantly higher than that required for kojic acid. Although the anti-tyrosinase abilities of the CC-EO and its major constituent, cinnamaldehyde, were significantly less than that of kojic acid, it was evident that they did have potent tyrosinase inhibitory activity.

Kinetic analyses confirmed that both CC-EO and *trans*-cinnamaldehyde were regarded as mixed-I type inhibitors for the enzyme with L-DOPA as the substrate. This result implies that CC-EO and *trans*-cinnamaldehyde affected the affinity of the enzyme for L-DOPA but did not bind at the active site (Webb 1963). Furthermore, the behavior of mixed-I type inhibition indicated that CC-EO and *trans*-cinnamaldehyde could bind, not only with the free enzyme, but also with the enzyme-substrate complex. As shown in Table 2, the determined values showed that the *K*_i_ value for *trans*-cinnamaldehyde was approximately 2 times lower than that of CC-EO. In other words, *trans*-cinnamaldehyde had a more effective binding capacity for the enzyme than CC-EO. Additionally, the value of *αK*_i_ was almost 4 times as great as *K*_i_ for the oxidation of L-DOPA, indicating that the affinity of the inhibitor, CC-EO or *trans*-cinnamaldehyde, for a free enzyme is stronger than that of inhibitor for the enzyme–substrate complex. Lee et al. (2000) reported that *trans*-cinnamaldehyde isolated from the bark of *C. cassia* exhibited competitive inhibition for L-DOPA oxidation by mushroom tyrosinase, while Lee (2002) reported that cinnamaldehyde isolated from the root of *P. cernua* was a noncompetitive tyrosinase inhibitor. However, different enzyme preparation and assay methods or inhibitor purities could have contributed to these differences in enzyme inhibitory kinetics (Chen et al. 1991b).

Recently, safe tyrosinase inhibitors have become important for their potential applications in improving food quality and preventing pigmentation disorders and other melanin-related human health issues (Fitzpatrick et al. 1983; Seo et al. 2003). Furthermore, safe tyrosinase inhibitors are important in cosmetics for skin whitening effects. *C. cassia* has been safely used for many years by humans as a flavoring agent and a traditional medicinal herb. According to the United States Food and Drug Administration (USFDA), *Cinnamomum* spp., including common and cassia cinnamon, are generally recognized as safe (GRAS) when used in amounts commonly found in food (Dugoua et al. 2007). Furthermore, cinnamaldehyde and benzaldehyde, which are found in CC-EO, are also GRAS (Kubo and Kinst-Hori 1999). Thus, it is expected that CC-EO and its major constituent, cinnamaldehyde, may be a safe and viable source of skin-whitening agents.

## Conclusions

In summary, our findings demonstrate that the CC-EO and its major component, cinnamaldehyde, possess potent inhibitory activity against the diphenolase activity of tyrosinase. A study of the kinetics for the inhibition of mushroom tyrosinase showed that the CC-EO and cinnamaldehyde are mixed-type inhibitors for the enzyme with L-DOPA as the substrate. Both CC-EO and cinnamaldehyde are generally recognized as safe (GRAS). Therefore, CC-EO and cinnamaldehyde may be suggested as a safe and as a good source of skin-whitening agents for pharmaceutical and cosmetic applications.

## Authors’ original submitted files for images

Below are the links to the authors’ original submitted files for images. Authors’ original file for figure 1 Authors’ original file for figure 2 Authors’ original file for figure 3
