# Supplementary figures and images for: Chemical composition and tyrosinase inhibitory activity of Cinnamomum cassia essential oil
Source: Bot Stud. 2013 Aug 21;54:10. doi: 10.1186/1999-3110-54-10 (PMC5432840; doi:10.1186/1999-3110-54-10)

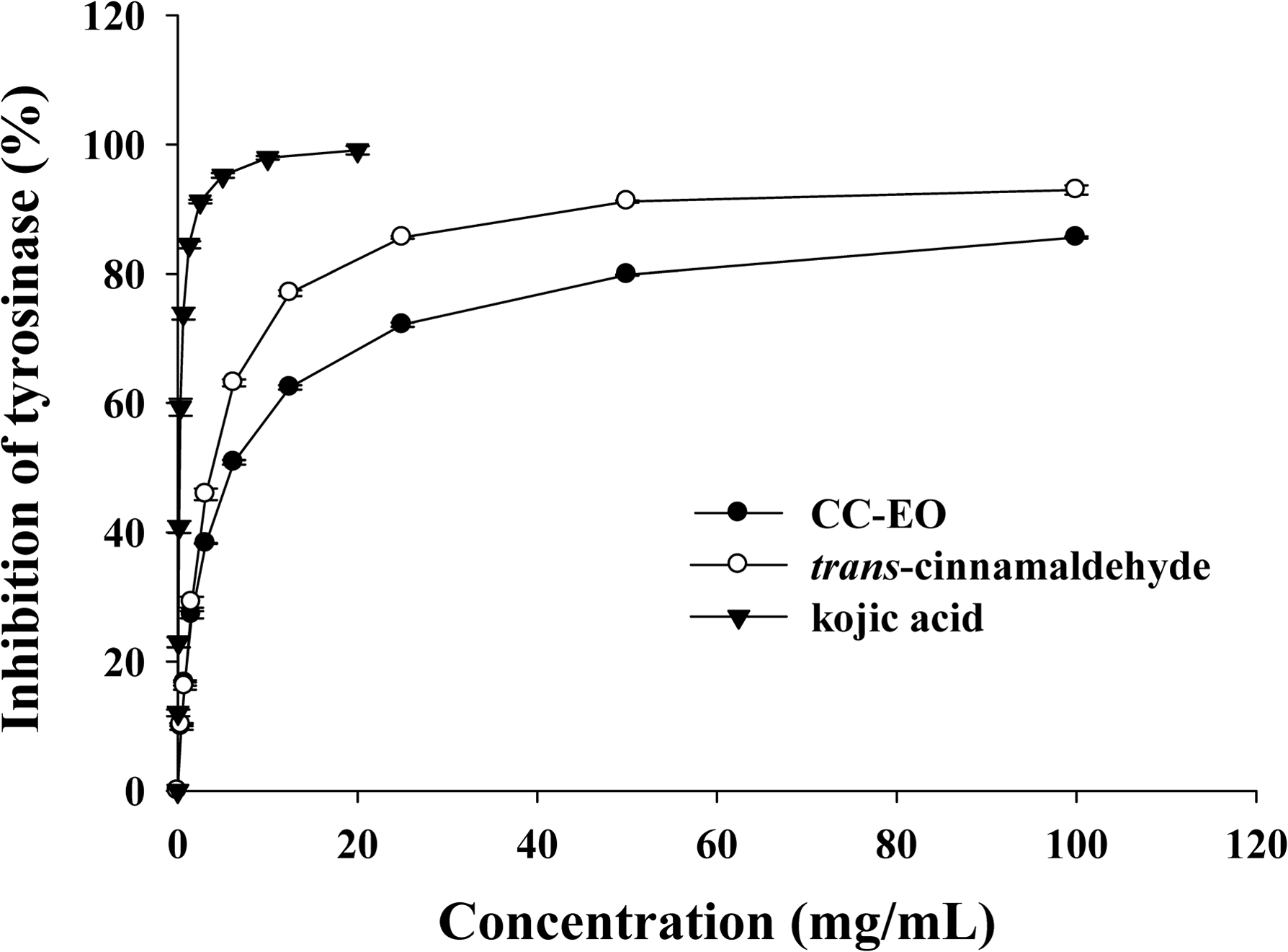

Supplement: Supplementary file 1 — Authors’ original file for figure 1 [file 40529_2012_7_MOESM1_ESM.tif]

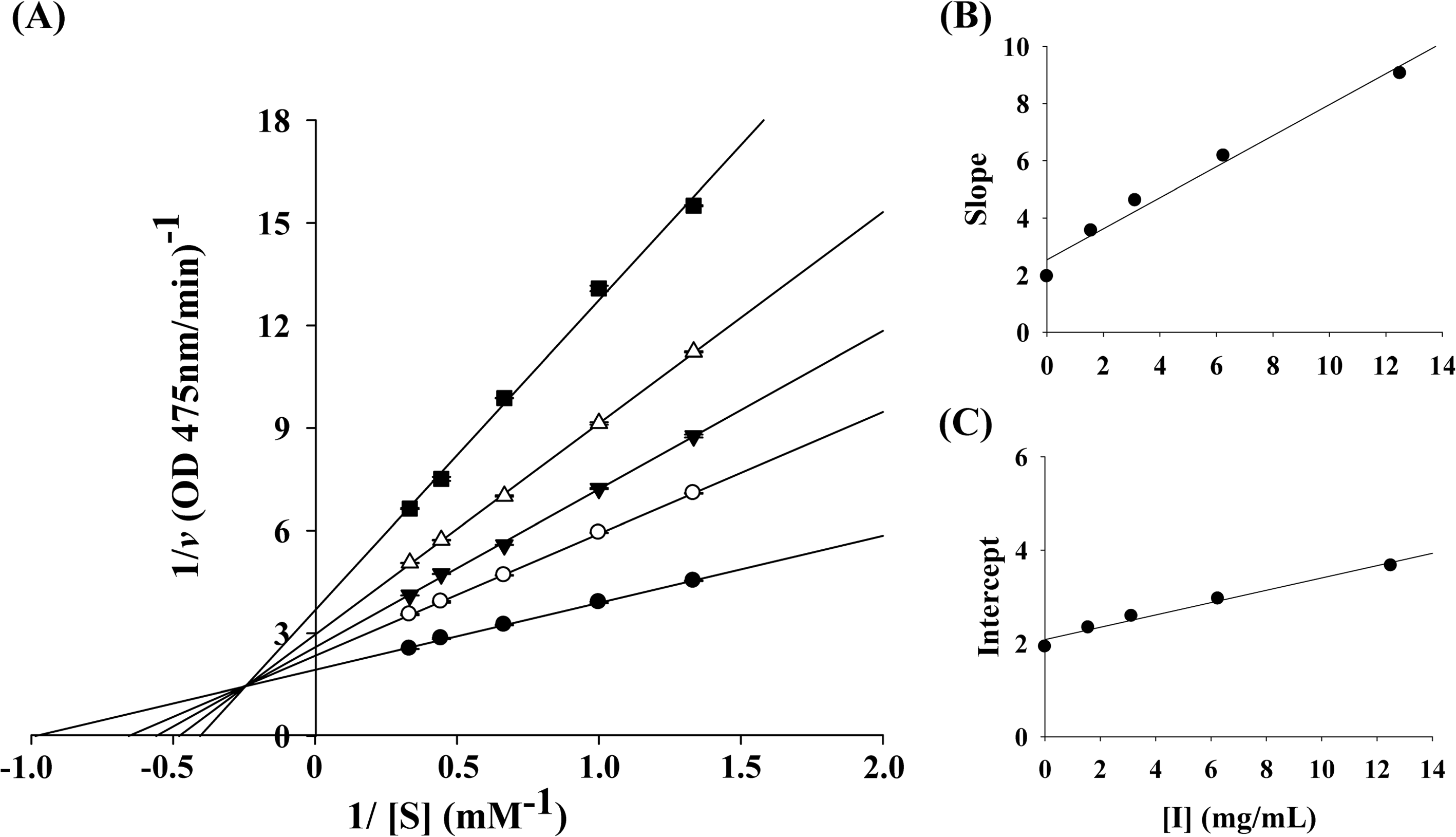

Supplement: Supplementary file 2 — Authors’ original file for figure 2 [file 40529_2012_7_MOESM2_ESM.tif]

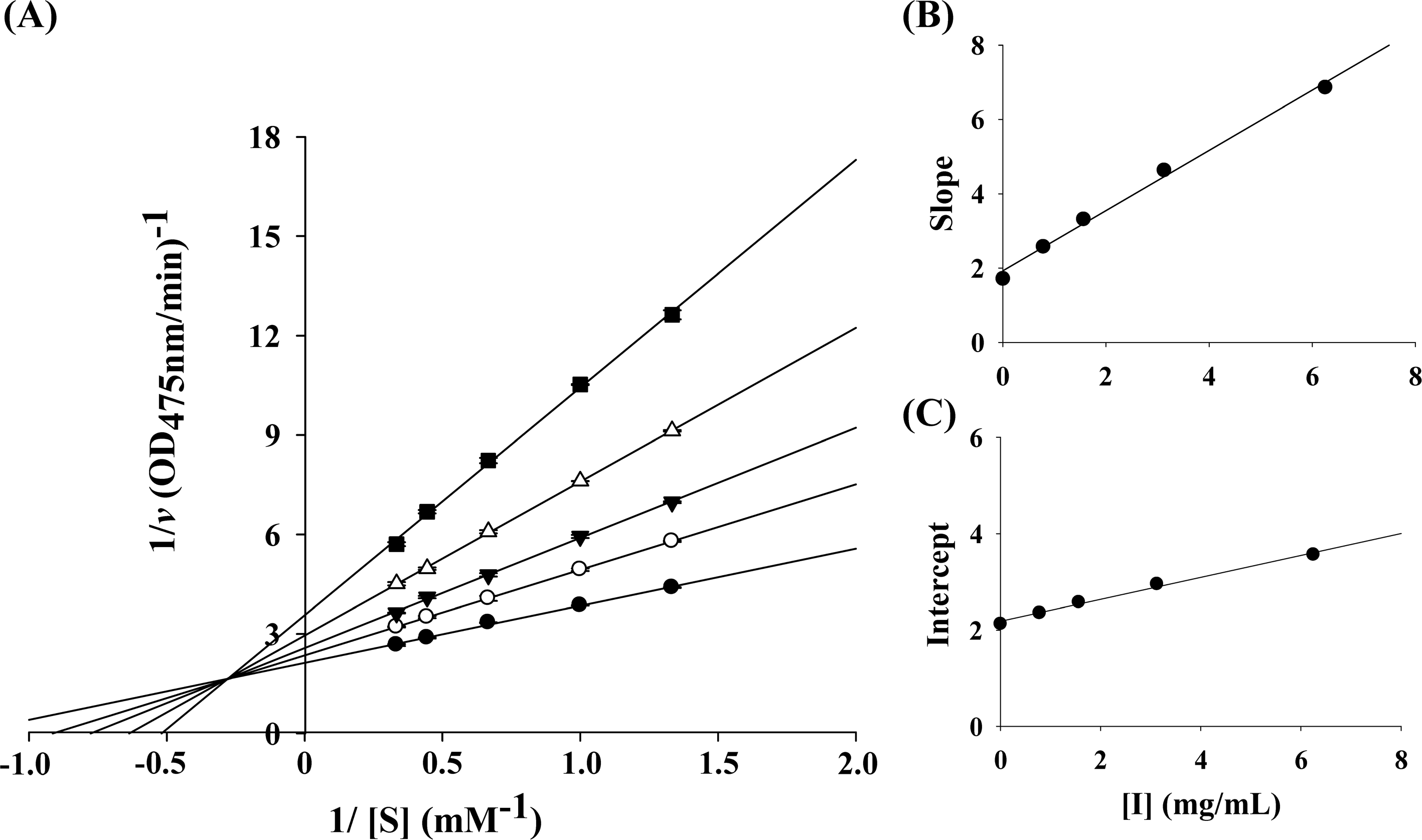

Supplement: Supplementary file 3 — Authors’ original file for figure 3 [file 40529_2012_7_MOESM3_ESM.tif]
